# Estimating transportation role in pandemic diffusion in Nigeria: A consideration of 1918-19 influenza and COVID-19 pandemics

**DOI:** 10.7189/jogh.10.020501

**Published:** 2020-12

**Authors:** Olabisi Michael Olapoju

**Affiliations:** Department of Geography, Obafemi Awolowo University, Ile-Ife, Nigeria

## Abstract

**Background:**

The focus of the study is to assess the role of different transport means in the importation and diffusion of 1918-19 influenza and a novel 2019 corona virus designated as COVID-19 in Nigeria.

**Methods:**

The study provides a review of the means by which the two pandemics were imported into the country and the roles the transport means of each period played in the local spread of the epidemics.

**Results:**

The study notes that seaports and railways, being the emerging transportation modes in the country were significant to the importation and local diffusion of 1918-19 influenza, respectively, while air transport is significant to the importation of the current COVID-19 pandemic.

**Conclusions:**

The study concludes that increasing preference for the transport at a given epoch is significant to the diffusion of prevailing epidemic in the epoch.

For most of human history, population have been largely relatively dispersed both at regional and continental scales. However, recent centuries have witnessed an extensive human contact as a result of development in means of transportation, accompanying trade expansion, population boom and city expansion. While development in transport technology till date continues to enhance peoples’ socio-economic and political existence, movement of disease has also been associated with increasing human movement through development in transportation network. Specifically, records of disease diffusion in the last 500 years had provided numerous examples of how the establishment and expansion of worldwide transport networks has facilitated global pandemics of communicable diseases. For instance, the Black Death of 14th Century revealed accounts of transmission and eventual death that was consistent with the arrival of travellers in Sicily in 1347 [1] and whose further transmission was halted by limiting transportation and movement patterns. There was the 1817 Cholera outbreak in India which later became global pandemic as it spread across many countries of the world along trade routes reflecting the expanding reach of the global transport system and increased movement of people [2]. The influenza pandemic of 1918-19 presented one of the world’s most devastating short-term demographic disasters, killing an estimated 40 million people in about 12 months globally [3,4]. The speed of transmission of this infectious disease was greatly influenced by growing transport network in the world.

Exactly one century away, at the end of 2019, an infectious disease known as coronavirus named as SARS-CoV-2) by International Committee on Taxonomy of Viruses (ICTV) and now tagged COVID-19 (arising from the family of the virus and the year of emergence) broke out in Wuhan, China. Since discovery, and as at the time of writing, COVID-19 has spread to over 214 countries and territories with a total case of 1438994 confirmed cases and 85 586 deaths globally. High transmission efficiency of COVID-19 is attributed to convenience of global travel. The objective of this study is to provide an assessment of the nature and role of transport in the importation, traffic and spread of 1918-19 influenza and COVID-19 in Nigeria. This is with a view to examining changing significance associated with prevailing transportation at the period of epidemics. The study reviews historical data of 1918-19 Influenza in Nigeria as well as report of cases of current pandemic known as COVID-19 in the country. Next sections provide review of the 1918-19 Influenza in Nigeria, situation of COVID-19 in Nigeria, comparative narration of the nature and role of transport in diffusion of 1918-19 Influenza and COVID-19 in Nigeria, discussion of the comparative review and conclusion.

## 1918-1919 INFLUENZA IN NIGERIA

The influenza pandemic of 1918-19 was one of the most pervasive and devastating biological disasters ever recorded in the world. Though there were contentions on the origins of 1918-19 influenza pandemic, its spread however, was typical of that of human travel, as it traveled from place to place. Major diffusion began in Europe to the rest of the world. Africa recorded its first hit from a ship that had docked in a British port from where it carried persons infected by influenza to Freetown, Sierra Leone in August 1918 [5]. In a similar vein, an American vessel *S.S. Shonga,* which made a brief stop in Freetown carried influenza victims in Freetown to Cape Coast. The ship which anchored for a few days in Cape Coast continued its journey to Accra with virtually all its crew down with influenza. However, by September 14, 1918, the influenza hit Lagos through Lagos seaports by an ocean liner, *S.S*. *Bida* which carried already infected passengers from Accra, Gold Coast, who, on arrival in Lagos passed the disease to Lagos residents. Inhabitants around the seaports of Marina and Apapa, especially seamen working on ships docked on harbour ports were one of the first sets of people to be infected with this virus. The epidemic spread quickly into the hinterland especially Lagos mainland and then followed trade routes, such as railway lines, motor roads, rivers and caravan routes. The progress of spread of the epidemic was based on the speed of normal transport prevailing on each highway [6]. But since the train was the major means of local transportation back then, locations such as Abeokuta, Ibadan, Illorin, Bida, Jebba, Zaria, Kano, and Bauchi which were linked to the existing rail lines were quickly affected by the epidemic [7]. While populations along the Western railway continued to be stricken by this disease, passenger vessels continued to bring infected people into other coastal towns of Nigeria. For instance, by 28 September, another vessel called *S.S. Batanga* had arrived Calabar port with a man suspected to be a victim of influenza. Also, Forcados was not spared as ocean liner *S.S. Ravenston* brought crew and passengers already infected by the disease to Forcados on 27 September. Other ports affected included Burutu, Warri, Port-Harcourt and Bonny with this epidemic penetrating various residential districts and neighbouring towns by roads. By December 1918, it had spread all over the country right from the first outbreak in Lagos.

## CASES OF COVID-19 IN NIGERIA

By the end of 2019, an infectious disease known as coronavirus named as SARS-CoV-2) by International Committee on Taxonomy of Viruses (ICTV) and now tagged COVID-19 (arising from the family of the virus and the year of emergence) broke out in Wuhan, China. By January 30, 2020, WHO (World Health Organization) declared COVID-19 epidemic as a public health emergency of global concern [8]. According to WHO report on 19th March, 2020, there are 209839 confirmed cases of COVID-19, 8778 confirmed deaths across 169 countries of the world. As at the time of writing this manuscript, there was a global record of 1 438 994 cases of infection and over 85 586 deaths across over 214 countries and territories of the world [8]. Though intermediate source of origin of this disease as well as clinically approved drug or vaccine have not been determined, there is a clear evidence that it assumes high human-to-human (hence, h2h) transmission mode. In addition, international travelling was adjudged to have heightened the importation risk of this virus especially from the affected locations in China thus making most countries vulnerable to the epidemic. More vulnerable is African continent because China at present is Africa’s leading commercial partner as large travel volumes through which the epidemic could reach the continent already exist. However, Africa recorded its first case in Egypt on February 14, 2020 through travellers returning from hotspots in Asia, Europe and the United States [8]. Since then, more than 50 countries have reported cases, initially, mainly confined to capital cities, and now spreading to a multiple provinces. By February 27, 2020, Nigeria’s first case was recorded when a 44-year Italian citizen was diagnosed of COVID-19 in Lagos State. The infected man arrived at the Murtala Muhammed International Airport, Lagos at 10pm on 24th February 2020 aboard Turkish airline from Milan, Italy. He traveled on to his company site in Ogun state on 25th February. On 8th March 2020, one of the asymptomatic contacts to the index case in Ogun was confirmed positive. On 17th March 2020, a 30-year old Nigerian national was diagnosed of COVID-19 in Lagos. She returned from the United Kingdom on the 13th of March. On the 18th of March 2020, five new confirmed cases of COVID-19 recorded in Nigeria, bringing the record to 8 cases. All the new five cases had a travel history to the UK or USA; four were detected in Lagos while one detected in Ekiti had contact with a traveler from USA. On the 19th of March 2020, four new confirmed cases of COVID-19 recorded in Nigeria bringing the recorded cases to twelve confirmed cases. All the four new cases were from Lagos. One had a travel history to the UK; one to France, 3^rd^ case was a contact to one of the previously confirmed cases; 4^th^ case had no history of travel but lived with foreigners/expatriates. On the 21^st^ of March 2020, three new confirmed cases of COVID-19 were recorded in Nigeria. Two of the three new cases had travel history to the United Kingdom and United States of America. One case had contact with recent travelers. On the 22^nd^ of March 2020, five new confirmed cases of COVID-19 were recorded in Nigeria. Four of the five new cases had travel history to high risk countries. The fifth case was a contact of a confirmed case. On the 23^rd^ of March 2020, ten new confirmed cases of COVID-19 were recorded in Nigeria with the first death. Eight of the ten new cases had travel history to high risk countries. As at the time of writing, Nigeria had a record of 305 confirmed cases and 8 deaths (**Table 1**).

**Table 1:** Periodic situation report of COVID-19 in affected States in Nigeria*

S/no	States	Total confirmed cases	Total confirmed death	Total discharged cases
1	Lagos	163	2	46
2	FCT	56	2	7
3	Osun	20	-	1
4	Edo	12	1	-
5	Oyo	11	-	-
6	Ogun	7	-	2
7	Bauchi	6	-	-
8	Kaduna	6	-	-
9	Akwa-Ibom	5	-	-
10	Katsina	4	1	-
11	Kwara	2	-	-
12	Delta	2	1	-
13	Enugu	2	-	-
14	Ekiti	2	-	1
15	Rivers	2	-	1
16	Ondo	2	-	-
17	Benue	1	-	-
18	Niger	1	-	-
19	Anambra	1	-	-
**Total**		**305**	**7**	**58**

*Source: [9].

## COMPARATIVE NARRATION OF THE NATURE AND ROLE OF TRANSPORT IN DIFFUSION OF 1918-19 INFLUENZA AND COVID-19 IN NIGERIA

Historical data has provided evidence of the role of transport, cross-border movement and regional flow of humans as veritable means of disease importation and spread. Assessment of 1918-19 influenza and COVID-19 revealed that water transport (ocean and inland waterways) and air transport played significant gateway roles in the importation of the two pandemics. Whereas, railway and road ways played significant role in lateral spread of both pandemics. The evolution and growth of Nigeria’s seaports belong to contrasting historical periods – the pre-modern and modern periods [10]. These periods were made up of some phases of alternating port concentration or spatial consolidation and port diffusion or dispersed port development in Nigeria. The interest of this study is the period of port development that coincided with the outbreak of 1918-19 influenza in Nigeria. This period was between 1910-1950, the fifth stage of development and a period of port concentration. At this period, Lagos, Akassa, Old Calabar, Sapele, Warri and Degema ports had become the dominant ports in the hierarchy of all the ports in Nigerian space economy. Specifically, in 1912, Lagos port had become more dominant handling about 41% of the total port traffic in the country, while Burutu and Calabar ports were handling 15% and 11% of total traffic respectively. The significance of this was seen in the diffusion of 1918-19 influenza through these ports. It is noteworthy that concentration of Nigerian trade in Lagos ports, Burutu, Calabar, Warri, Port-Harcourt and Bonny ports were responsible for the penetration of the epidemics into the country. However, with the import of the epidemic into the national territory, local transmission took its turn with rail, road and inland water transports playing significant roles in the diffusion of the epidemic locally.

The significance of rail transport in internal spread of 1918-19 influenza in Nigeria was apparent because it coincided with the periods when network of railways were being constructed across the country between 1898 and 1965 [11] primarily to facilitate economic exploitation and administrative control of their regions. The raging of the influenza along the railway lines was such as if railway network was planned in preparation for the epidemic. Railway influence on local transmission of the epidemic was underscored by the report of the occurrence of the epidemic in virtually all Northern towns and villages, commencing with settlements along Ilorin-Kano railway, with Kano becoming primary point of diffusion in Northern part of Nigeria. The influenza moved southward along the course of Niger River. By October 14, the epidemic was brought to Onitsha through Lokoja and later spread to entire towns and villages [7]. The spread continued eastward to reach Owerri, Okigwe and Enugu-Ngwo and many other towns along the Eastern Railway. The influence of rail transport was also significant in the spread of the epidemic to Benin Kingdom, though this region was also adjudged to be bedeviled from coastal area of Forcados, Warri and Sapele.

However, with severe damage that was done to transport infrastructure during the Civil War coupled with the post reconstruction and building preference given to road ways over railways, as well as perennial operational and organizational challenges, preference for rail transport has reduced to its abysmal minimum and major traffic greatly reduced. Since then, Nigeria railway remains static in structure and highly dormant to emerging transport dynamics across the globe in the last 3 to 4 decades, especially considering modernization, and advancement in railway infrastructure and equipment which have enabled railway fulfill its role efficiently and effectively. Though recently, governments have mustered some efforts at revamping the railway system in some parts of the country, its present state may account for its non-significance to local transmission of recent epidemic in the country.

On the contrary, air transport plays the most significant role in the importation of COVID-19 in Nigeria as is the case with virtually all the countries that are affected by the epidemic. The significance of air transport in today’s global endeavours is imprinted in most socio-economic calculations, political negotiations and environmental considerations in all of the world’s deliberations. This is reflective of Barnes’ prediction of air transport becoming a vital component of the economies of major cities, as it provides fast personal access for business, social, or recreational purposes, as well as fast physical access to resources and markets [12]. It is informing to note that air transport is yesterday’s future transport. The role played in the diffusion of COVID-19 is unavoidably significant, especially considering the humongous passenger traffic it handles in recent times. According to Airports Council International (ACI), global airports handled 5.4 billion passengers in 2011 [13] and this is expected to be more than double, exceeding 12 billion by 2031 [14]. Over the past few decades, air transport networks have developed and internationalized though very unevenly, overall technological development, internationalization of economic development, increasing purchasing power, increasing tourism activities among other issues have led to sharp rise in air connections both at regional and international scales. It is thus significant to note that attendant asymbiotic relationship exists between global ease of travel provided by air transport and the spread of epidemic and disease. The ease with which inter-regional and inter-continental travel is been achieved in the recent period is *sine qua non* to the ease of importation of COVID-19.

## DISCUSSION

The role of transportation in the diffusion of epidemics has not been unnoticed at different times of outbreak of epidemics. This study assessed the significance of mode of human transportation to importation and local diffusion of two epidemics which occurred century apart—1918-19 influenza and current COVID-19 epidemics. The study observed that though both epidemics have not their origin in Nigeria, the first case recorded for each of the pandemic were both imported into the country through Lagos State which provides a veritable gateway to Nigerian economy. However, while the 1918-19 influenza came through seaport, COVID-19 came through airport. Both presenting major international assess to the country. Each pandemic reflects the expanding reach and efficiency of the global transport system and increased movements of people [2]. For instance, the significance of seaports in the importation of 1918-19 influenza was based on the development of seaports in the country which provided the only option for international movement as pioneer commercial air transport which was to provide a new way of moving goods and passengers around the earth did not start until 1920s—a period after the epidemic. Whereas, the efficiency, speed and reach of air transport networks was responsible for the importation of COVID-19, a completely new disease. This is supporting the view of ever-expanding air transport network as significant to the extent and speed of spread of disease [15-17]. Thus, the prominence gained by air transport in the importation of COVID-19 above seaports as an alternative gateway to Nigerian economy suggests that the transport of the future has significant role to play in the diffusion of the disease of the future.

Though, the study highlighted the role of railway in the intra-national diffusion of 1918-19 influenza, it is not certain it has any role in local spread of COVID-19. This may be due to very low traffic patronizing rail transport because of its dormancy for a very long time until recent time when attention is being paid to its resuscitation. It may also be due to the suspension of rail transport activities by Nigerian Railway Corporation (NRC) (Independent News Online, the 21^st^ March 2020) as a way of checking the spread of COVID-19.

## CONCLUSION

1918-19 influenza and COVID-19 happened a century apart with both having debilitating impact on human health with attendant death records. The significance of transportation in the importation and local transmission of the two epidemics were obvious. It was evident that the efficiency of spread of each epidemic was determined by the origin of the epidemic and efficiency of the prevailing transport means. It thus means that future transport may have significant influence on the efficiency with which likely future epidemic or disease will be diffused.

The limitation of this study is that while one of the epidemics considered had happened about a century ago, the other epidemic is still very much around and fresh with us. Thus, current figures on the confirmed cases, death and discharge cases will be needed for further study. Also similar study may be carried out to look into a comparative study of the distinctive role of existing transport means in the importation and diffusion of epidemics in sub-Saharan African countries as a whole.
